# Genotype-Phenotype Correlations in Neurofibromatosis Type 1: A Single-Center Cohort Study

**DOI:** 10.3390/cancers13081879

**Published:** 2021-04-14

**Authors:** Marcello Scala, Irene Schiavetti, Francesca Madia, Cristina Chelleri, Gianluca Piccolo, Andrea Accogli, Antonella Riva, Vincenzo Salpietro, Renata Bocciardi, Guido Morcaldi, Marco Di Duca, Francesco Caroli, Antonio Verrico, Claudia Milanaccio, Gianmaria Viglizzo, Monica Traverso, Simona Baldassari, Paolo Scudieri, Michele Iacomino, Gianluca Piatelli, Carlo Minetti, Pasquale Striano, Maria Luisa Garrè, Patrizia De Marco, Maria Cristina Diana, Valeria Capra, Marco Pavanello, Federico Zara

**Affiliations:** 1 Department of Neurosciences, Rehabilitation, Ophthalmology, Genetics, Maternal and Child Health (DINOGMI), University of Genoa, 16132 Genoa, Italy; marcelloscala87@gmail.com (M.S.); cristinachelleri@libero.it (C.C.); andreaaccogli@gaslini.org (A.A.); riva.anto94@gmail.com (A.R.); v.salpietro@ucl.ac.uk (V.S.); bocciardi@unige.it (R.B.); paolo.scudieri@unige.it (P.S.); minettic@unige.it (C.M.); strianop@gmail.com (P.S.); federico.zara@unige.it (F.Z.); 2 Pediatric Neurology and Muscular Diseases Unit, IRCCS Istituto Giannina Gaslini, University of Genoa, 16147 Genoa, Italy; giangi.piccolo@gmail.com (G.P.); gmorcaldi@fastwebnet.it (G.M.); monicatraverso@gaslini.org (M.T.); mcristinadiana@gaslini.org (M.C.D.); 3 Department of Health Sciences, Section of Biostatistics, University of Genova, 16132 Genoa, Italy; irene.schiavetti@gmail.com; 4 UOC Genetica Medica, IRCCS Istituto Giannina Gaslini, University of Genoa, 16147 Genoa, Italy; francescamadia@gaslini.org (F.M.); marcodiduca@gaslini.org (M.D.D.); francescocaroli@gaslini.org (F.C.); simonabaldassari@gmail.com (S.B.); m.iacomino87@gmail.com (M.I.); PatriziaDeMarco@gaslini.org (P.D.M.); valeriacapra@gaslini.org (V.C.); 5 Neuro-Oncology Unit, IRCCS Istituto Giannina Gaslini, 16147 Genova, Italy; antonioverrico@gmail.com (A.V.); claudiamilanaccio@gaslini.org (C.M.); mluisagarre@gaslini.org (M.L.G.); 6 UO Dermatologia, IRCCS G. Gaslini, 16147 Genoa, Italy; gianmariaviglizzo@gaslini.org; 7 Neurosurgery Department, IRCCS Istituto Giannina Gaslini, University of Genoa, 16147 Genoa, Italy; gianlucapiatelli@gaslini.org

**Keywords:** neurofibromatosis type I, *NF1*, NGS, cDNA sequencing, MLPA, genotype–phenotype correlations, brain tumor, splicing, stop-gain

## Abstract

**Simple Summary:**

Neurofibromatosis type 1 (NF1) is a complex disorder characterized by a multisystem involvement and cancer predisposition. It is caused by genetic variants in *NF1*, a large tumor suppressor gene encoding a cytoplasmatic protein (neurofibromin) with a regulatory role in essential cellular processes. Genotype–phenotype correlations in NF1 patients are so far elusive. We retrospectively reviewed clinical, radiological, and genetic data of 583 individuals with at least 1 National Institutes of Health (NIH) criterion for NF1 diagnosis, including 365 subjects fulfilling criteria for the diagnosis. Novel genotype–phenotype correlations were identified through uni- and multivariate statistical analysis. Missense variants negatively correlated with neurofibromas. Skeletal abnormalities were associated with frameshift variants and whole gene deletions. The c.3721C>T; p.(R1241*) variant positively correlated with structural brain alterations, whereas the c.6855C>A; p.(Y2285*) variant was associated with a higher prevalence of Lisch nodules and endocrinological disorders. These novel NF1 genotype–phenotype correlations may have a relevant role in the implementation of patients’ care.

**Abstract:**

Neurofibromatosis type 1 (NF1) is a proteiform genetic condition caused by pathogenic variants in *NF1* and characterized by a heterogeneous phenotypic presentation. Relevant genotype–phenotype correlations have recently emerged, but only few pertinent studies are available. We retrospectively reviewed clinical, instrumental, and genetic data from a cohort of 583 individuals meeting at least 1 diagnostic National Institutes of Health (NIH) criterion for NF1. Of these, 365 subjects fulfilled ≥2 NIH criteria, including 235 pediatric patients. Genetic testing was performed through cDNA-based sequencing, Next Generation Sequencing (NGS), and Multiplex Ligation-dependent Probe Amplification (MLPA). Uni- and multivariate statistical analysis was used to investigate genotype–phenotype correlations. Among patients fulfilling ≥ 2 NIH criteria, causative single nucleotide variants (SNVs) and copy number variations (CNVs) were detected in 267/365 (73.2%) and 20/365 (5.5%) cases. Missense variants negatively correlated with neurofibromas (*p* = 0.005). Skeletal abnormalities were associated with whole gene deletions (*p* = 0.05) and frameshift variants (*p* = 0.006). The c.3721C>T; p.(R1241*) variant positively correlated with structural brain alterations (*p* = 0.031), whereas Lisch nodules (*p* = 0.05) and endocrinological disorders (*p* = 0.043) were associated with the c.6855C>A; p.(Y2285*) variant. We identified novel NF1 genotype–phenotype correlations and provided an overview of known associations, supporting their potential relevance in the implementation of patient management.

## 1. Introduction

Neurofibromatosis type 1 (NF1, MIM #162200), also known as Von Recklinghausen disease, is a very complex though relatively common genetic condition characterized by a heterogeneous involvement of several organ systems [1,2]. This multifaceted disorder is caused by autosomal dominantly inherited or de novo pathogenic variants in *NF1* (MIM *613113), a large tumor suppressor gene located at 17q11.2 and spanning ~350 kb of genomic DNA sequence [1]. The most abundant *NF1* transcript consists of 57 exons (NM_000267.3) and encodes the prevalent isoform of neurofibromin (NP_000258.1), a 2818-amino acids multidomain protein acting as a main regulator of the RAS signaling pathway and playing a pivotal role in the regulation of essential cellular processes [3,4,5]. The constitutional haploinsufficiency due to germinal *NF1* loss-of-function variants causes the more common generalized form of NF1, whereas somatic variants arising during fetal development may result in segmental/mosaic NF1, in which clinical manifestations are limited to the mutated tissues [6].

The pleiotropy of *NF1* and the complex pathophysiology mechanisms involved in most NF1-related clinical manifestations underlie the typical largely variable clinical expressivity, which may occur even within the same family for a specific *NF1* variant [7]. However, well-defined clinical hallmarks are recognizable, including distinctive pigmentary defects of the skin (café-au-lait macules—CALMs—and intertriginous freckling, especially axillary and inguinal) and the eye (hamartomas of the iris, known as iris Lisch nodules), as well as typical tumors of the peripheral nervous system (PNSTs) such as cutaneous/subcutaneous (cNFs/sNFs) and plexiform neurofibromas (pNFs) [6,8,9]. Additional relevant clinical features are optic pathway gliomas (OPGs) and manifestations of bone dysplasia (e.g., sphenoid wing dysplasia, tibial dysplasia, and thinning of long bone cortex) [6,8,9]. According to the National Institutes of Health (NIH) Consensus Development Conference diagnostic criteria, NF1 diagnosis can be established in an individual presenting with ≥2 of these clinical manifestations, with positive family history considered equivalent to a clinical criterium [10,11]. Although their presence is not critical for diagnosis, craniofacial features (macrocephaly and hypertelorism), vascular abnormalities (e.g., renal artery stenosis, cardiovascular abnormalities, and cerebral vasculopathy), endocrinological disorders (e.g., hyperthyroidism or endocrine tumors), and neuroimaging abnormalities (unidentified bright objects—UBOs—and cerebral malformations) are also clinically relevant [12,13,14]. The multisystem involvement may lead to numerous clinical symptoms, such as psychomotor delay, learning disabilities, behavioral disturbances, epilepsy, neurological deficits, short stature, scoliosis, hormonal imbalances, and hypertension [6]. Eventually, a phenotypic overlap with Noonan syndrome may occur in some patients presenting with a distinctive NF1 clinical variant known as neurofibromatosis-Noonan syndrome (NFNS) [15].

NF1 is the most common tumor-predisposing disease and affected individuals usually develop characteristic PNSTs, including aggressive malignancies such as malignant peripheral nerve sheath tumors (MPNSTs). Furthermore, there is a higher susceptibility to several types of tumors, especially those involving the central nervous system (CNS) (e.g., gliomas other than OPGs), endocrine tissues (e.g., pheochromocytoma and carcinoid) or musculoskeletal system (rhabdomyosarcoma) [16,17]. NF1 has also been associated with an increased risk of breast cancer, with specific *NF1* variants conferring a distinctive cancer risk [18]. In general, major concerns consist of brain tumors in childhood and MPNSTs in the third and fourth decades of life [19]. The main underlying mechanism in NF1-related PNST is the loss of both functional *NF1* alleles due to a somatic second hit (via loss of heterozygosity—LOH, somatic intragenic mutations, or, very rarely, promoter hypermethylation) [20,21]. Interestingly, the same mechanism is involved in the pathogenesis of nontumoral manifestations such as CALMs [22]. NF1-related cancerogenesis is complex and the contribution of additional genetic alterations is usually crucial for the development of MPNSTs and brain tumors [6]. Specific *NF1* variants predict a distinctive risk for malignancies and the molecular signature of NF1-associated tumors is different from sporadic neoplasms [23]. Eventually, the malignant transformation of tumors in NF1 patients also involves epigenetic and microenvironmental factors [23].

The multisystem functional relevance of *NF1* and the correlated variable clinical expressivity in NF1 patients make it difficult to establish clear-cut genotype–phenotype correlations. However, aside from cancer risk, distinctive clinical or neuroimaging features have been associated with specific *NF1* variants [24,25]. For example, severe clinical manifestations were linked to missense variants affecting the codons 844–848, whereas a Noonan-like phenotype has been recently associated with missense variants affecting p.Met1149, p.Arg1276, and p.Lys1423 residues [26,27].

In this study, we explored genotype–phenotype correlations in an NF1 population including both pediatric and adult cases. We retrospectively reviewed clinical charts, imaging findings, and genetic testing results from a large cohort of 583 individuals displaying at least 1 NIH diagnostic criterion. Subsequently, we focused on the subpopulation of subjects with NF1 diagnosis according to the NIH criteria (i.e., fulfilling ≥ 2 criteria). After delineating the distribution of the predominant clinical features in the population, we analyzed their association with *NF1* variant types through uni- and multivariate analysis. We also investigated the presence of statistically significant correlations between recurrent *NF1* variants and specific clinical and neuroimaging features. Eventually, based on the analysis of genetic testing results in patients fulfilling or not NIH criteria for diagnosis, we discussed the emerging need for an update of current NF1 diagnostic criteria in light of a greater role of genetic findings.

## 2. Results

### 2.1. Study Population

The target population consisted of 583 individuals of different ancestries (Albanian, American, Brazilian, Bulgarian, Chinese, Congolese, Egyptian, Filipino, French, Georgian, German, Indian, Italian, Macedonian, Moldavian, Moroccan, Nigerian, Romanian, Senegalese, Spanish, Sri Lankan, Turkish, Ugandan, Ukrainian, and Saudi) and presenting with at least one NIH criterion for NF1 diagnosis (Table S1). The patients were referred to the Medical Genetics Unit of IRCCS Giannina Gaslini (Genoa, Italy) for diagnostic purposes between 2009 and 2020.

The age of enrolled subjects ranged from 1 to 73 years (mean 14) (Table 1), with 407 patients (69.8%) being in the pediatric age group (≤18 years).

Among the 583 patients, 309 were males and 274 females (male/female sex ratio 1.13), whereas in the pediatric group males were 224 and females 183 (ratio 1.22). All patients are currently alive except three of them, deceased at the age of 8 (#83), 16 (#250), and 12 years (#290), respectively. The deceased/alive ratio was 0.005 in the whole cohort and 0.007 for pediatric patients. A positive family history of NF1 (first-degree relative meeting the NIH diagnostic criteria) was ascertained in 134/583 (22.9%) cases, including 83/407 (20.4%) pediatric patients.

Two or more NIH diagnostic criteria were fulfilled in 365 individuals (62.6%) (group A), 235 of whom aged less than 18 years (64.4%). In this group, males were 182/365 (49.9%) and females 183/365 (50.1%), with a male/female sex ratio of 0.99. Among the 235 pediatric patients, males were 120 and females 115 (male/female sex ratio 1.04). The deceased patients (#83, #250, and #290) belong to this subpopulation, with general and pediatric deceased/alive ratios of 0.008 and 0.0128, respectively. A positive family history was present in 104/365 (28.5%) cases, including 66/235 (28.1%) pediatric patients.

Differences in the baseline characteristics emerged from the comparison between groups A (patients with at least ≥2 NIH criteria) and B (patients not fulfilling NIH criteria), also in relation to genetic diagnosis (Table 1). In the B group, the mean age was lower (12.5 versus 15 years, *p* < 0.001), with a prevalence of subjects aged 0–12 years (50% versus 37.8%, *p* = 0.009), and males were predominant (58.3% versus 49.9%, *p* = 0.049). A higher percentage of positive family history (28.5% versus 13.8%, *p* = 0.001) and a larger number of NIH criteria met (3 versus 1, *p* < 0.001) were instead observed in the A group. A genetic diagnosis was more frequently achieved in the A group (78.6% versus 29.4%, *p* < 0.001), with a higher prevalence of maternally inherited variants (8.2% versus 4.1%, *p* < 0.001). Accordingly, patients with a definite genetic diagnosis similarly had a higher prevalence of positive family history (30.8% versus 11.2%, *p* < 0.001) and met a larger number of NIH criteria (3 versus 1, *p* < 0.001).

### 2.2. Phenotypic Characterization

**Figure 1:**
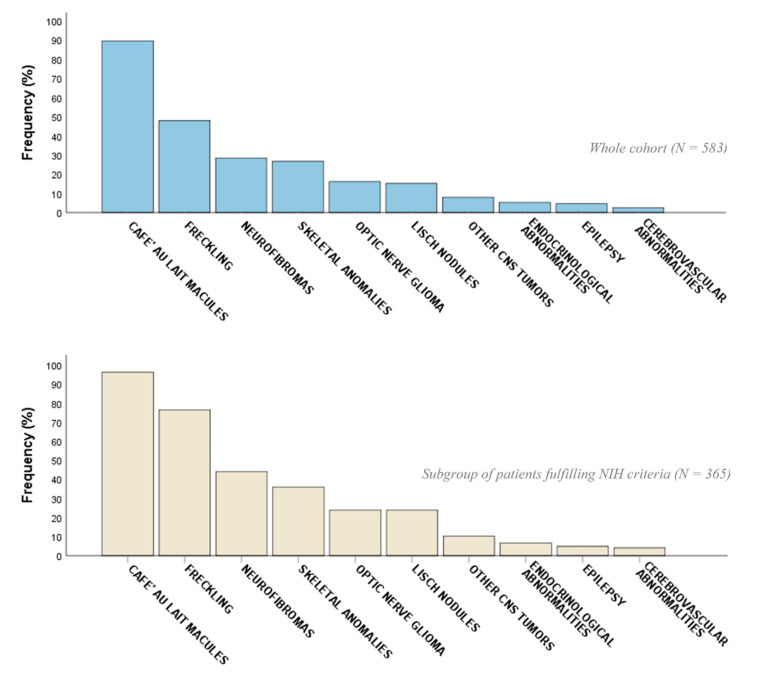
Percentage distribution of the frequency of the most common NF1-related clinical features in the whole cohort of patients with at least 1 NIH criterion (*n* = 583) and in the subpopulation fulfilling NIH criteria for NF1 diagnosis (group A, *n* = 365).

Skin manifestations were the most common clinical features (Figure 1), with CALMs and freckling being identified in 522/583 (89.5%) and 280/583 (48%) cases, respectively. In the A group, CALMs were observed in 351/365 (96.2%) and freckling in 279/365 (76.4%) individuals, respectively.

Neurofibromas were observed in 213/583 (36.5%) individuals, with cNFs/sNFs being diagnosed in 137/583 (23.5%) and pNFs in 76/583 (13%) subjects, respectively. In the A group, 209 subjects were diagnosed with neurofibromas (57.3%), including 134 with cNFs/sNFs (36.7%) and 75 with pNFs (20.5%) (Table 2).

Among NIH criteria, Lisch nodules were diagnosed in 89/583 (15.3%) cases, sphenoid bone dysplasia in 15/583 (2.6%), and tibial dysplasia in 13/583 (2.2%). In the A group, these features were observed in 87/365 (23.8%), 14/365 (3.8%), and 11/365 (3%) individuals, respectively.

A variable neurological involvement was observed in 249/583 (42.7%) individuals. Common neurological features (headache, epilepsy, behavioral abnormalities, severe learning disabilities, and developmental delay/intellectual disability—DD/ID) were found in 120/583 (20.6%) subjects, of whom 59 (10.1%) also displayed additional neurological manifestations (abnormal muscle tone, abnormal deep tendon reflexes, ataxia, tremor, fatigue, lower limbs pain, hallucinations, neurogenic bladder, hyperkinetic movements, paresthesias, and stereotyped movements). Of note, the latter were present alone in 129/583 (22.1%) subjects. Epilepsy occurred in 27/583 (4.6%) cases, headache compromising normal functioning in 27/583 (4.6%), severe learning disabilities in 17/583 (2.9%), behavioral abnormalities (autism spectrum disorder—ASD, attention deficit hyperactivity disorder—ADHD, and avoidant/restrictive food intake disorder—ARFID) in 12/583 (2.1%), and severe DD/ID in 12/583 (2.1%). In the A group, neurological involvement was present in 177/365 (48.5%) subjects, with common neurological features in 85/365 (23.3%) cases and additional neurological findings alone or in combination with these features in 92/365 (25.2%) and 44/365 (12.1%) individuals, respectively. Eighteen subjects had epilepsy (4.9%) and 23 headache (6.3%). Severe learning disabilities, behavioral abnormalities, and DD/ID were observed in 14/365 (3.8%), 12/365 (3.3%), and 7/365 (1.9%) individuals, respectively.

Overall, CNS tumors occurred in 119/583 (20.4%) individuals and OPGs were the most common (94/119, 79%). Less frequent tumors included other brain gliomas (e.g., pilocytic astrocytoma, other astrocytomas, and glioblastoma) (24/119, 20.2%), non-glioma brain tumors (amartoma, meningioma, and lipoma) (24/119, 20.2%), and spinal tumors (astrocytomas) (3/119, 2.5%). Of note, 25/119 (21%) individuals were diagnosed with more than one CNS tumor. As to extra-nervous tumors, patient #257 was diagnosed with pheochromocytoma (1/583, 0.17%) and patients #110 and #135 with multiple lipomatosis (2/583, 0.34%). In the A group, CNS tumors occurred in 103/365 (28.2%) patients, of whom 24 (23.3%) had more than one CNS tumor. OPGs were the most common (87/103, 84.5%), followed by other gliomas (22/103, 21.4%), non-glioma brain tumors (15/103, 14.6%), and spinal tumors (2/103, 1.9%). Subjects #110, #135, and #257 belonged to this group, leading to a 0.82% rate for extra-nervous tumors.

Skeletal abnormalities were observed in 155/583 (26.6%) cases, including scoliosis (89/583, 15.3%), pectus excavatum (10/583, 1.7%), sphenoid bone dysplasia (15/583, 2.6%), tibial dysplasia (13/583, 2.2%), and other skeletal alterations (osteoporosis, vertebral defects, lower limbs dysmetria, and craniosynostosis) (55/583, 9.4%). More than one skeletal feature was present in 14/155 (9%) subjects. In the A group, skeletal involvement was present in 131/365 (35.9%) individuals, of whom 24 (18.3%) with more than one skeletal alteration. Skeletal features included scoliosis (77/365, 21.1%), pectus excavatum (10/365, 2.7%), sphenoid bone dysplasia (14/365, 3.8%), tibial dysplasia (11/365, 3%), and other skeletal alterations (44/365, 12.1%).

Among the other multisystem clinical features, endocrinological abnormalities (thyroid dysfunction, Addison’s disease, and precocious puberty) were diagnosed in 31/583 (5.3%) cases, vascular and lymphatic disorders (angioma, cavernoma, and lymphatic dysplasia) in 11/583 (1.9%), and renal involvement (malformations, hydronephrosis, and renovascular disease) in 8/583 (1.4%). Cardiovascular involvement was present in 13/583 (2.2%) cases, in the form of hypertension (7/13, 54%), pulmonic stenosis (PS) (3/13, 23%), or other cardiac conditions (arrythmias and valvulopathies) (3/13, 23%). Less common were Noonan-like dysmorphic features (NLDFs) (13/583, 2.2%) and ocular abnormalities (glaucoma and coloboma) (4/583, 0.7%). In the A group, endocrinological abnormalities were diagnosed in 24/365 (6.6%) subjects, vascular and lymphatic disorders in 8/365 (2.2%), and renal alterations in 4/365 (1.1%). Cardiac involvement was present in 10/365 (2.7%) subjects, including hypertension (6/10, 60%), PS (2/10, 20%), and other cardiac disorders (2/10, 20%). NLDFs and ocular abnormalities were observed in 8/365 (2.2%) and 3/365 (0.8%) patients, respectively.

Structural brain lesions were found in 36/583 (6.2%) subjects, including Chiari II malformation (#104), tethered cord (#99), syringomyelia (#30 and #561), and meningocele (#104 and #173). Additional neuroimaging findings were corpus callosum hypoplasia (#409), dural ectasia (#303), cerebellar hypoplasia (#69), olfactory bulb hypoplasia (#340), and cerebral atrophy (#137). Cerebrovascular involvement was present in 26/583 (4.5%) cases, with more than one cerebrovascular alteration in 10/583 (1.7%). Moyamoya syndrome (MMS) was diagnosed in 15/583 (2.6%) patients, variants in cerebrovascular anatomy in 7/583 (1.2%), and other abnormalities (aneurysms, ectasias, and hypoplasia) in 15/583 (2.6%). In the A group, neuroimaging abnormalities were present in 29/365 (7.9%) patients and cerebrovascular involvement in 25/365 (6.8%) subjects, including MMS (14/365, 3.8%), variants in cerebrovascular anatomy (7/365, 1.9%), and other abnormalities (15/365, 4.1%).

In the A group, there was a higher prevalence of skin manifestations (CALMS, 96.2% versus 78.4%, *p* < 0.001; freckling, 76.4% versus 0.5%, *p* < 0.001), neurofibromas (cNFs/sNFs, 36.7% versus 1.4%, *p* < 0.001; pNFs, 20.5% versus 0.5%, *p* < 0.001), gliomas (OPG, 23.8% versus 3.2%, *p* < 0.001; gliomas other than OPG, 6% versus 0.9%, *p* = 0.008), Lisch nodules (23.8% versus 0.9%, *p* < 0.001), skeletal features (scoliosis, 21.1% versus 5.5%, *p* < 0.001; sphenoid bone dysplasia, 3.8% versus 0.5%, *p* = 0.026; pectus excavatum, 2.7% versus 0%, *p* = 0.013; other skeletal alterations, 12.1% versus 5%, *p* = 0.005), and cerebrovascular involvement (MMS, 3.8% versus 0.5%, *p* = 0.026; other cerebrovascular abnormalities, 4.1% versus 0%, *p* = 0.005). Neurological involvement was also more common, both in the form of common neurological features (in general, 23.3% versus 16.1%, *p* = 0.032; headache, 6.3% versus 1.8%, *p* = 0.012; behavioral abnormalities, 3.3% versus 0%, *p* = 0.007) and other neurological findings (37.3% versus 23.9%, *p* = 0.001).

Patients with a genetic diagnosis showed a higher frequency of freckling (63.2% versus 25%, *p* < 0.001), neurofibromas (cNFs/sNFs, 30.8% versus 12.5%, *p* < 0.001; pNFs, 19.4% versus 3.4%, *p* < 0.001), gliomas (OPG, 20.5% versus 9.5%, *p* = 0.009; gliomas other than OPG, 6% versus 1.3%, *p* = 0.018), Lisch nodules (21.1% versus 6.5%, *p* < 0.001), skeletal features (scoliosis, 17.9% versus 11.2%, *p* = 0.027; pectus excavatum, 2.6% versus 0.4%, *p* = 0.05), and NLDFs (3.4% versus 0.4%, *p* = 0.017). In these subjects, common neurological features (23.6% versus 15.9%, *p* = 0.024), behavioural abnormalities (3.1% versus 0.4%, *p* = 0.024), and other neurological findings (37% versus 25%, *p* = 0.002) were also more common.

### 2.3. Genetic Data Analysis

A definite genetic diagnosis could be achieved in 351/583 (60.2%) individuals. More in detail, a causative *NF1* alteration was identified in 287/365 (78.6%) subjects belonging to the A group versus 64/218 (29.4%) individuals from the B group (Table 1). Single nucleotide variants (SNVs) were detected in 326/583 (55.9%) subjects, including 267/365 (73.2%) from the A group and 59/218 (27.1%) from the B group (Table 3).

Among the 351 subjects with genetic confirmation, copy number variations (CNVs) were detected in 25 (7.1%) cases (Table 3). In particular, CNVs were identified in 25/233 (10.7%) subjects in whom Multiplex Ligation-dependent Probe Amplification (MLPA) assay was performed. Of these rearrangements, 20 (80%) were detected in the A group (20/102 patients tested, 19.6%) and 5 (20%) in the B group (5/131 patients tested, 3.8%). In the pediatric age group, the overall diagnostic rate was 57%, with 214/407 (52.6%) individuals harboring a SNV and 18/407 (4.4%) carrying a CNV. In the A group, a genetic diagnosis was achieved in 189/235 (80.4%) pediatric patients. Causative SNVs were detected in 175/189 (92.6%) and CNVs in 14/189 (7.4%) subjects, with a CNVs diagnostic rate of 23.3% (60 patients tested with MLPA). Interestingly, likely pathogenic/pathogenic SNVs and CNVs were detected in 64 individuals from the B group, of whom 43 (67.2%) were pediatric patients (39 SNVs and 3 CNVs) and 21 (32.8%) adults (20 SNVs and 1 CNV).

**Figure 2:**
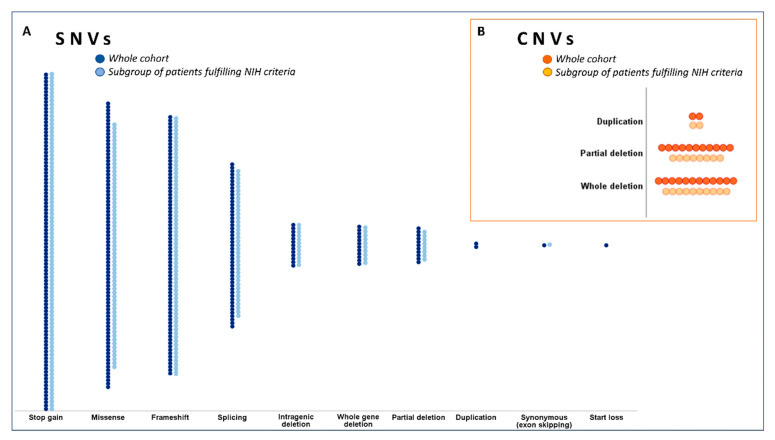
Genetic results: distribution of *NF1* variants. (**A**) Types and frequency distribution of the single nucleotide variants (SNVs) detected in the population of subjects meeting at least 1 diagnostic NIH criterion (*n* = 583) and in the group of patients meeting ≥ 2 NIH criteria (group A, *n* = 365). (**B**) Types and frequency distribution of the detected copy number variations (CNVs) among the 233 individuals tested with MLPA in the whole cohort and the 102 individuals tested in the group A.

Pathogenic SNVs (Figure 2A) encompassed several variant types (missense, splicing, stop-gain, start loss, synonymous, frameshift, and small intragenic deletion).

Among the SNVs, the most common were stop-gain variants (100/326, 30.7%), followed by missense (85/326, 26.1%), frameshift (77/326, 23.6%), and splicing (49/326, 15%) variants. All the identified variants are rare (allele frequency < 0.001) or absent in the gnomAD database (https://gnomad.broadinstitute.org, accessed on 7 January 2021). Aside from clearly loss-of-function variants (stop-gain, frameshift, and splicing variants), all missense variants are predicted to negatively affect protein function by in silico tools (SIFT, Poly-Phen2, and CADD) (Table S2).

Pathogenic CNVs (*n* = 25) consisted of 2 partial gene duplications (8%), 11 partial gene deletions (44%), and 12 whole gene deletions (48%) (Figure 2B). Of these, 18 rearrangements were detected in the pediatric age group: 2 duplications (11.1%); 7 partial deletions (38.9%); 9 whole gene deletions (50%). The duplications were multi-exon, involving exons 2–8 (#350) or 37–47 (#351), whereas partial deletions involved several distinct exons, alone or in combination. The most commonly affected exons (deleted in at least 3 patients) were exon 1, 38, and 39. Single exon deletions were detected in exons 1 (#329, #330, and #349), 3 (#10), and 6 (#331). The largest deletion involved instead exons 9–46 (#332), but other multi-exon deletions were also identified (e.g., exons 31–57 deletion in #335 and exons 52–58 deletion in #333). In the A group, 20 CNVs were detected: 8 partial deletions (40%), 10 whole gene deletions (50%), and 2 duplications (10%). Among these, 14 (63.6%) were carried by pediatric patients.

**Figure 3:**
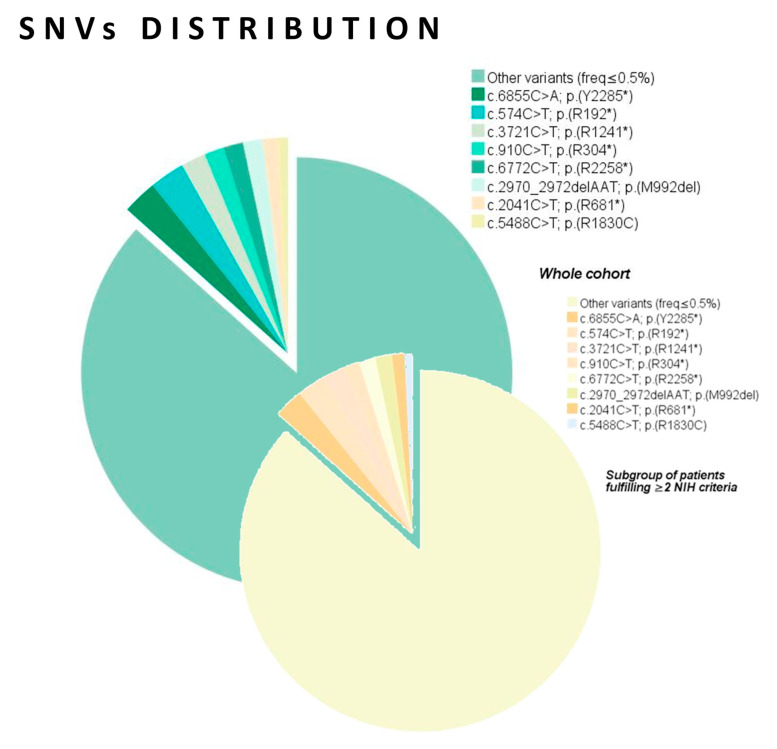
Pie chart showing the distribution of the recurrent *NF1* variants in the whole cohort (*n* = 583) and the subgroup of patients fulfilling ≥2 NIH criteria (group A, *n* = 365).

The most frequent *NF1* variants in the A group (according to the NM_001042492.3 transcript, NP_001035957.1) were c.574C>T; p.(R192*) in seven cases; c.6855C>A; p.(Y2285*) in seven cases; c.3721C>T; p.(R1241*) in five cases; c.5488C>T; p.(R1830C) in five cases; c.6772C>T; p.(R2258*) in four cases; c.910C>T; p.(R304*) in four cases; c.2041C>T; p.(R681*) in four cases; c.2970_2972delAAT; p.(M992del) in four cases (Table 3). These variants were identified in distinct, unrelated index cases. The frequency of the remaining variants was <0.5% (Figure 3).

### 2.4. Genotype–Phenotype Correlations

Genotype–phenotype correlations were specifically investigated in the subpopulation of patients fulfilling NIH diagnostic criteria (group A). The multi-variate analysis revealed statistically-significant correlations for freckling, neurofibromas (cNFs/sNFs/pNFs), Lisch nodules, skeletal alterations, structural brain lesions, and endocrinological abnormalities.

Freckling was less commonly observed in patients with a positive NF1 family history (OR = 0.41; 95%CI = 0.23–0.73; *p* = 0.003) (Table 4).

The risk of neurofibromas (pNFs) was lower in subjects with missense variants (OR = 0.28; 95%CI = 0.11–0.67; *p* = 0.005) (Table 5).

The presence of Lisch nodules was higher in subjects harboring the c.6855C>A; p.(Y2285*) variant compared to those with different *NF1* variants (OR = 6.03; 95%CI = 0.98–36.94; *p* = 0.05), but lower in patients with a positive family history (OR = 0.40; 95%CI = 0.21–0.78; *p* = 0.007) (Table 6).

The presence of skeletal alterations positively correlated with whole gene deletions (OR = 4.09; 95%CI = 0.98–17.08; *p* = 0.05) and frameshift variants (OR = 2.81; 95%CI = 1.34–5.87; *p* = 0.006) (Table 7).

The presence of structural brain lesions on brain magnetic resonance imaging (MRI) (e.g., meningocele, syringomyelia, and Chiari II malformation) did positively correlate with the stop-gain variant c.3721C>T; p.(R1241*) (OR = 7.65; 95%CI = 1.21–48.36; *p* = 0.031) (Table 8).

The risk of endocrinological disorders was higher in patients in whom the c.6855C>A; p.(Y2285*) variant was detected (OR = 5.82; 95%CI = 1.06–32.13; *p* = 0.043) (Table 9).

There was a negative association between the NF1 missense variants and the presence of cNFS and sNFs (OR = 0.44; 95%CI = 0.22–0.90; *p* = 0.024) (Table 10).

Of note, neurofibromas were more common among patients older than 13 years, which is in line with their natural course in NF1 [6]. However, a statistically significant association could be only confirmed for the age range 13–18 years at the multivariate analysis (OR = 3.36; 95%CI = 1.91–5.91; *p* <0.001). Eventually, no relevant correlation could be identified for common neurological features (Table S3), OPG (Table S4), scoliosis (Table S5), CALMs (Table S6), and other neurological findings (Table S7).

## 3. Discussion

The clinical manifestations of NF1 are largely heterogeneous, making it an extremely proteiform condition, with a few peculiar cardinal features playing the pivotal role in the diagnostic process [7]. According to the 1987 NIH criteria, the diagnosis is mainly clinical and can be established in an individual presenting with two or more of the following features: ≥6 CALMs; intertriginous freckling; OPG; ≥2 Lisch nodules; ≥2 neurofibromas of any type or ≥1 pNF; peculiar skeletal alterations (sphenoid dysplasia or tibial pseudoarthrosis); positive family history (first degree relative with NF1) [9,10]. The frequency of these main features in the NF1 populations is variable, also depending on their differential age at onset/diagnosis and sensitivity of the clinical assessment [28,29]. An essential contribution to the overall increase in NF1 diagnostic rate has been certainly provided by genetic testing [30]. Although the clinical diagnosis can be established in subjects showing the classic NF1 phenotype, molecular genetic testing may still reveal fundamental to establish a diagnosis in patients with suggestive clinical features but not strictly fulfilling the NIH criteria [31,32]. Genetic testing also has relevant implications for the counseling of first-degree relatives (e.g., recurrence risk, family planning, and prenatal testing) [33]. Eventually, the emerging field of genotype–phenotype correlations offers new insights into the complex pathophysiology of NF1 and may significantly impact the clinical management and prognosis of affected individuals [34]. Of note, NF1 highly variable clinical expressivity is also determined by genetic modifiers not directly linked to the *NF1* locus and allelic heterogeneity of constitutional *NF1* variants [34,35,36].

The first genotype–phenotype correlation was observed in patients harboring *NF1* deletions. Microdeletions occur in 5–10% of NF1 patients and not infrequently involve neighboring genes. These rearrangements lead to a more severe “contiguous gene syndrome” with higher incidence of neurofibromas and MPNSTs, likely attributable to the involvement of tumor suppressor genes in co-deleted regions (e.g., *SUZ12*—OMIM *606245, *RNF135*—OMIM *611358, and *ADAP2*—OMIM *608635) [37,38,39,40]. Learning disabilities are also common [24,41]. Interestingly, not only large deletions, but also truncating and splicing *NF1* variants were associated with complex clinical phenotypes "requiring medical attention" [24]. More than 2800 different SNVs in *NF1* have been identified, including only 31 recurrent variants [26]. The first SNV to be associated with a specific clinical phenotype was the c.2970_2972delAAT, p.(Met992del) (NM_001042492.3). This 3-bp inframe deletion in exon 17 was detected in patients with a milder clinical phenotype, lacking cNFs/sNFs/pNFs but typically showing learning difficulties and cognitive impairment [27,42]. Around 1% of NF1 patients harbor missense variants affecting the Arg1809 (Arg1830 according to the NM_001042492.3 transcript) [43]. These subjects show CALMs and NLDFs, but they lack neurofibromas and other typical NF1 features [43,44,45]. A similar phenotype has been reported in patients harboring the c.3112A>G, p.(Arg1038Gly) variant (NM_001042492.3) [46]. An enrichment of inframe variants in exon 24 of *NF1* and missense variants involving Met1149, Arg1276, and Lys1423 have been also observed in patients with Noonan-like features [15,27]. Of note, variants in Met1149 cause a mild clinical phenotype (pigmentary manifestations without nervous tumors), symptomatic spinal neurofibromas are associated with Arg1276 variants, and a higher frequency of cardiovascular abnormalities is observed in subjects haboring variants in Arg1276 and Lys1423 [27]. A severe clinical presentation positively correlated with missense variants affecting one of the five neighboring codons Leu844, Cys845, Ala846, Leu847, and Gly848 in the cysteine-serine-rich domain (CSRD) of NF1 [26]. The most recurrent variants were the c.2540T>C, p.(Leu847Pro) and c.2542G>C, p.(Gly848Arg) (NM_001042492.3) [26]. In addition to a high incidence of skeletal abnormalities, patients had a high predisposition to develop malignancies and a higher incidence of pNFs, spinal neurofibromas, and OPGs [26]. As to NF1-related multisystem features, SNVs localized to the 5′ tertile of *NF1* and affecting residues 1-909 (including CSRD) have been associated with a higher incidence of short stature, especially loss-of-function variants [47]. A higher prevalence of non-truncating intragenic variants was observed in patients with congenital heart defects (CHD), with a two- and six-fold higher risk of developing CHD and PS, respectively [48].

Genotype–phenotype correlations also impact the cancer risk and the overall prognosis of NF1 patients. Frameshift variants are the most common SNVs and usually occur de novo, likely favored by the high mutation rate of *NF1* and possible modifying factors [49,50]. Of note, truncating variants are associated with elephantiasis neuromatosa and malignancies (MPNSTs, breast cancers, and gastrointestinal stromal tumors—GIST) [51]. Splicing variants account for ~30% of all SNVs [52]. A higher incidence of tumors of the nervous system (especially brain gliomas and MPNSTs) and connective tissues was observed in patients harboring splicing variants, supporting a focused clinical surveillance in these subjects and a wider application of RNA-based screening analyses [53]. Recent re-analysis did not confirm the association between OPGs and the clustering of variants in the 5′ tertile of *NF1* (especially exons 4b and 10a) or in the CSRD, supporting the lack of specific genotype–phenotype correlations [54,55,56,57]. The c.6007-5A>G and c.6263T>C; p.(Leu2088Pro) variants (NM_001042492.3) were detected in a subgroup of individuals with multiple spinal tumors, a condition known as “spinal fibromatosis” (SF) [34,58,59]. Non-coding RNAs (ncRNAs) act as transcriptional and post-transcriptional regulators of gene expression, and their deregulation may play a relevant role in cancerogenesis [60]. Interestingly, a specific genotype of the long-ncRNA *ANRIL* rs2151280 correlated with the development of OPGs in mildly affected subjects, possibly representing a diagnostic and prognostic marker [60]. Eventually, *NF1* variants were found to correlate with volumetric imaging features of neurofibromas depending on their type and localization [25].

Some of the previously reported genotype–phenotype associations were confirmed in our cohort. The c.2970_2972delAAT, p.(Met992del) variant was detected in four patients (#139, #143, #144, and #145) with a milder phenotype mainly consisting of CALMs, freckling, Lisch nodules (#144), and NLDFs (#143) [27,42]. Neurological involvement was present (#143, #144, and #145), but without severe cognitive impairment. Of note, cNFs/sNFs were diagnosed in patient #143, gliomatosis cerebri in #143, and OPG in #144. Six patients harbored variants affecting Arg1809. The p.(Arg1830Cys) variant (p.(Arg1809Cys) according to the NM_000267.3 transcript) was detected in subjects #234, #235, #236, #237, and #238. The p.(Arg1830Pro) (p.(Arg1809Pro) according to the NM_000267.3 transcript) was detected in patient #239. In line with previous reports, these patients presented with CALMs and freckling, whereas a neurological involvement was only present in #234 and #235 [43]. Interestingly, patient #236 was diagnosed with cNFs/sNFs, which are occasional in patients harboring variants in Arg1809 [43]. The p.(Arg1038Gly) variant was detected in a patient with CALMs (#150), confirming the lack of other NF1-related features associated with this specific change [46]. Patient #130 harbored the p.(Ala846Pro) variant, affecting one of the five codons (844–848) associated with elevated incidence of skeletal features and higher cancer risk [26]. Accordingly, this patient showed skeletal abnormalities, whereas no malignancies were diagnosed.

Novel statistically-significant genotype–phenotype correlations also emerged. With regard to the type of *NF1* variation, we observed a higher frequency of skeletal abnormalities in patients harboring frameshift variants and whole gene deletions. Two specific stop-gain variants significantly correlated with distinctive NF1 features. The c.6855C>A; p.(Y2285*) variant was associated with a higher prevalence of Lisch nodules and endocrinological abnormalities, whereas the c.3721C>T; p.(R1241*) variant did positively correlate with the presence of structural brain lesions. However, the latter correlation needs further confirmation due to the heterogeneous nature of this clinical category. A novel negative correlation between missense variants and all types of neurofibromas (cNFs, sNFs, and pNFs) was also observed. Eventually, no significant correlation emerged for OPG, which is in line with the most recent observations [57].

Interestingly, six out of the eight recurrent variants identified in our cohort are stop-gain (c.574C>T; p.(R192*), c.6855C>A; p.(Y2285*), c.3721C>T; p.(R1241*), c.6772C>T; p.(R2258*), c.910C>T; p.(R304*), and c.2041C>T; p.(R681*)). All these variants are predicted to cause haploinsufficiency through the formation of a truncated transcript, likely leading to nonsense mediated mRNA decay (NMD). The remaining c.5488C>T; p.(R1830C) and c.2970_2972delAAT; p.(M992del) variants have different functional consequences. The missense variant p.(R1830C) falls within a 51 base-pairs mutational hot spot and affects a relevant functional domain (pleckstrin homologous domain—PH, which regulates the lipid binding in the adjacent Sec portion), likely leading to impaired protein function [1]. The p.(M992del) variant results instead in a NF1 protein lacking a very conserved Methionine in the CSRD, likely impacting on the function of this critical domain [42].

Recent surveys of NF1 cohorts have shown a variable distribution of *NF1* variant types, with some recurrent cardinal aspects [61,62,63,64]. In three studies, frameshift variants were the most common (20–45%), followed by splicing (25–30%), stop-gain (20–25%), missense (10–15%), and inframe indels (≤5%) variants [61,62,63]. Stop-gain variants were instead predominant (≃35%) in the study by Calì et al., followed by missense (≃30%), frameshift (≃20%), splicing (≃5%), and inframe indels (5%) variants [64]. Furthermore, in the Human Gene Mutation Database (HGMD, http://www.hgmd.cf.ac.uk/ac/index.php, accessed on 1 March 2021), *NF1* variant types distribution was: frameshift (≃45%); stop-gain (≃20%); missense (≃20%); splicing (15%); inframe indels (<5%) [62]. In our study, stop-gain variants were predominant (30.7%), followed by missense (26.1%), frameshift (23.6%), and splicing (15%) variants. These findings are in line with previous reports and the distribution observed in our cohort is comparable to that reported by Calì et al., aside from a higher prevalence of splicing variants (15% versus ≃5%) [61,62,63,64].

Interesting insights emerged from the comparison of the A and B groups for the distribution of baseline and phenotypic characteristics, also in relation to genetic diagnosis. The mean age of patients in the B group was lower, with a different distribution by age categories, regardless of genetic diagnosis. Although interesting, this finding is difficult to explain, since the mean age of these subjects is already beyond the threshold for the development of NF1 age-dependent manifestations [9]. In general, subjects with a genetic diagnosis met a larger number of NIH criteria (especially positive family history) and most of them were accordingly included in the A group. This finding is likely explained by the fact that more patients from this group attract the attention of clinicians and, therefore, undergo a genetic investigation. Interestingly, these subjects had a higher prevalence of maternally inherited variants in comparison to the B group. Aside from NIH criteria, some NF1-related features (gliomas other than OPG, scoliosis, pectus excavatum) and signs of neurological involvement were more common in the A group and in patients with genetic diagnosis. Additional clinical manifestations (cerebrovascular abnormalities, MMS, and other skeletal alterations) were also predominant in the A group, but regardless of genetic diagnosis. NLDFs were instead more frequent in subjects harboring causative *NF1* variants, regardless of the fulfillment of NIH criteria.

In our cohort, two subpopulations of NF1 patients raise intriguing food for thoughts due to their peculiar nature: (1) patients fulfilling NIH diagnostic criteria but lacking a genetic diagnosis; (2) patients with genetic diagnosis but not fulfilling NIH diagnostic criteria. The first subpopulation includes 78 cases (78/365 patients of the A group, 21.4%). MLPA assay was not performed in 17/78 (21.8%) patients, thus not excluding a possible causative CNV, but genetic testing through NF1 sequencing and MLPA was negative in 61/78 (78.2%) subjects. Although an unrevealed low-grade mosaicism cannot be ignored, in none of these 78 individuals clinical manifestations were suggestive of a mosaic NF1. Extensive NGS-based techniques (i.e., exome sequencing—ES—and genome sequencing—GS), will likely play a non-secondary role in the complex diagnostic process of these patients. The second subpopulation consists of 64 individuals (64/218 patients of the B group, 29.4%) not fulfilling NIH diagnostic criteria but harboring causative *NF1* variations, of whom 43/64 (67.2%) pediatric patients and 21/64 (32.8%) adult subjects. Clinical ascertainment biases (e.g., scarce experience in the identification of light freckling) and inappropriate instrumental investigations (e.g., lack of slit-lamp examination for the diagnosis of Lisch nodules) may certainly impair the detection of some NIH criteria. However, the not-negligible size of this subpopulation raises the question whether NIH criteria should be updated in light of a greater relevance of genetic findings (e.g., prompting a NF1 diagnosis in a subject with positive genetic testing and ≥1 NIH criteria), pointing towards a possible integration of genetic data in the NF1 diagnostic algorithm.

Our study has some limitations. Despite a thorough neuroimaging revision, the prevalence of UBOs and possible relative genotype–phenotype correlations could not be properly assessed, due to the lack of MRIs in some cases and the limited quality of available scans in some adult individuals. Another limitation is a possible bias in the clinical ascertainment of adult patients (e.g., unreliable stature assessment), which cannot be completely excluded. More in general, the enrollment of adult individuals might have slightly reduced the overall clinical sensitivity, as these subjects rarely undergo a periodic and complete medical checkup, unlike pediatric patients. This observation likely accounts for the lower-than-expected prevalence of some clinical features, such as the Lisch nodules. The lack of possible correlations involving CNVs is most likely explained by the limited number of patients harboring these rearrangements in the studied cohort. Eventually, nominal *p*-values were reported for all the investigated correlations, due to the exploratory nature of this analysis, which was not powered by a definite hypothesis [65,66]. In any case, the risk of false significance by multiplicity when performing multiple analysis is always considered in the discussion of results, also by reviewing the clinical plausibility and relevance of some results as well as the effect size of the correlations [66,67,68].

From a strictly biological perspective, some of the observed genotype–phenotype correlations suggest that very damaging genetic alterations (frameshift variants and whole gene deletions) might cause a tissue-specific effect (skeletal development abnormalities), likely explained by the dramatic impact of a complete loss of protein function in that tissue. Although dedicated functional studies are necessary to confirm this observation, the identification of other intriguing correlations hints at the possibility that poorly understood underlying genetic mechanisms are also involved. This is the case of the association of specific stop-gain variants with distinctive clinical features. Although different variant types are predicted to result in comparable negative functional consequences (e.g., stop-gain and frameshift variants are both predicted to cause a loss of function), diverse genotype-phenotype associations were observed for functionally equivalent variants. Even for a same variant type (stop-gain), two variants emerged for their positive correlation with peculiar and different clinical features. This is even more surprising as these variants are predicted to result in a complete loss of protein function through the same mechanisms, namely, NMD or generation of a truncated transcript. Most human genes generate different transcript isoforms arising from the use of alternative transcription start sites, polyadenylation sites, and splice sites [69]. These diverse transcripts are differentially expressed in a tissue- and time-specific manner, and the variation in the protein isoforms levels resulting from alternative transcript processing may act as a phenotypic modifier and contribute to NF1 phenotypic variability [69]. The negative correlation between missense variants and neurofibromas is especially interesting, suggesting a potentially lower impact of partial loss of function in the pathogenesis of these NF1 features as compared to complete protein deficiency. Of note, the biological consequences of missense variants can be extremely variable and even severe, depending on the affected residue and the functional domain involved. In light of the potential relevance of emerging NF1 genotype–phenotype correlations, these observations prompt the need to further explore the mechanisms underlying these associations through dedicated functional studies.

## 4. Materials and Methods

### 4.1. Phenotypic Characterization

The study was conducted in accordance with the ethical standards of the declaration of Helsinki. A retrospective review of the clinical charts of 583 patients fulfilling at least 1 NIH diagnostic criterion for NF1 was performed at the Department of Pediatric Neurology of the Giannina Gaslini Children’s Hospital. Imaging reports and, when available, original scans were thoroughly reviewed, including brain computed tomography (CT), brain and spinal MRI and magnetic resonance angiography (MRA), skeletal X-ray survey, abdominal ultrasonography, and echocardiograms. The results of additional diagnostic tests were also reviewed for phenotypic dissection (e.g., electrocardiogram, endocrine laboratory tests, and accurate dermatologic and ophthalmologic evaluations). Both clinical and imaging data were collected in a comprehensive database including the main NF1 phenotypic features based on their frequency and diagnostic relevance: cutaneous manifestations (CALMs, skin fold freckling), PNSTs (c/sNFs, pNFs), ophthalmologic findings (Lisch nodules), brain tumors (OPGs, gliomas other than OPG, other types of tumor), spinal tumors, neuroimaging abnormalities, skeletal abnormalities (scoliosis, short stature, pectus excavatum, tibial dysplasia, sphenoid bone dysplasia, other skeletal features), vascular alterations (cerebral vasculopathy, MMS, cerebrovascular anatomic variants, renal vasculopathy, lymphatic abnormalities), renal malformations, neurological symptoms (headache, epilepsy, behavioral abnormalities, learning disabilities, psychomotor delay, other neurological features), craniofacial features (macrocephaly, Noonan-like dysmorphic features), endocrinological abnormalities, and cardiovascular involvement (hypertension, PS, other cardiac alterations). For each patient, specific parameters were considered positive (indicated as 1), negative (indicated as 0), or not available (indicated as NA) (Table S1) when data were incomplete. Less common clinico-radiological findings were collected in a separate column of the database for each patient (clinical notes). Family history was reviewed in all cases and patients were evaluated as fulfilling or not NIH diagnostic criteria based on their overall clinical history and phenotype. For familial cases, only the probands (index cases) were included in the study and the causative NF1 variations were considered as single entries.

### 4.2. Genetic Testing

After informed consent was obtained from the parents or legal guardians, the patients underwent genetic testing. All enrolled subjects (*n* = 583) were screened for SNVs in *NF1* through Next Generation Sequencing (NGS) (*n* = 419) or direct sequencing of the complementary DNA (cDNA) (*n* = 164). The presence of CNVs was additionally investigated in a portion of the patients (*n* = 233) through the MLPA assay.

#### 4.2.1. NGS Assay Design, Library Preparation, and Sequencing

Written informed consent was obtained from all the patients’ parents or guardians. Genomic DNA was isolated from 1 mL of peripheral blood using QIAamp^®^ DNA Blood Midi (Qiagen, Courtaboeuf, Les Ulis, France). We assessed the concentration and the quality at 260/280 nm of the isolated DNA using the NanoDrop spectrophotometer (NanoDrop Technologies, DE, USA). *NF1* (57 exons, 8457 bp) and *SPRED1* (7 exons, 7780 bp) amplicons were designed using the AmpliSeq Designer Software v2.0 (Thermo Fisher Scientific, Carlsbad, CA, USA) targeting the coding sequences and 10 bases of the adjacent intron regions; the design resulted in a total of 136 amplicons divided in two multiplex primer pools.

Amplicon library was prepared using the Ion AmpliSeq Library Kit 2.0 (Thermo Fisher Scientific): the two primer pools were added to 10 ng of gDNA and amplified by PCR. The amplicons were ligated to the sequencing adapters, characterized by 20 different sequence-barcodes (Ion Xpress Barcode Adapters, Thermo Fisher Scientific) to allow sample assignment after pooling. The library was purified using the Agencourt AMPure XP Reagent (Beckmann Coulter, CA, USA). The concentration of the final library was determined by fluorescent measurement on Qubit 2.0 instrument (Life Technologies). Libraries from 20 different samples were diluted to 15 ng/mL and pooled together.

Template preparation was performed with Ion OneTouch kit v2 (Thermo Fisher Scientific) on Ion OneTouch2 instrument (Thermo Fisher Scientific) using an emulsion PCR method based on Ion Sphere Particle (ISP). The non-templated ISPs were removed during the semi-automated enrichment process on Ion ES instrument (Thermo Fisher Scientific). After adding the sequencing primer and polymerase (Ion PGM 200 Sequencing kit v2, Thermo Fisher Scientific), the fully prepared ISPs were loaded into an Ion 314 chip and the sequencing runs were performed with 500 flows on the Ion Torrent PGM machine (Thermo Fisher Scientific).

The sequence reads were analyzed using the Ion Reporter pipeline (Thermo Fisher Scientific) and the CLC Genomics Workbanch software (Qiagen, Courtaboeuf, France) for filtering out poor quality reads, alignment on hg19 reference, variant detection, and coverage analysis. Variant call was performed with a minimum coverage of 20 reads (≥20X). All the called variants were validated by Sanger sequencing. Sequence variant nomenclature was indicated according to HGVS (https://varnomen.hgvs.org, accessed on 21 January 2021).

#### 4.2.2. Multiplex Ligation-Dependent Probe Amplification (MLPA)

MLPA was performed using two commercial kits, the SALSA P081/P082 NF1 and the SALSA P295 SPRED1 kits (MRC-Holland, Amsterdam, The Netherlands). We used 100 ng of denatured genomic DNA from patients and control individuals in the overnight annealing of the exon-specific probes and subsequent ligation reaction. PCR was carried out with FAM-labeled primers using 10 μL of ligation reaction. Separation was performed using an ABI Prism 3100 Genetic Analyzer (Applied Biosystem, Foster City, CA, USA) and MLPA data analysis was done with the Coffalyser.Net™ v.1 software (MRC-Holland). A reduction or increase in the peak area values to <0.5 or >1.5 was considered an indication of a deletion or a duplication, respectively.

#### 4.2.3. RNA Extraction, RT-PCR

Peripheral blood was collected in PAXgene Blood RNA tubes and subjected to total RNA extraction using the PAXgene Blood RNA kit (Qiagen, Hilden, Germany) following the manufacturer instructions. RNA amount and quality were evaluated by NanoDrop (NanoDrop Technologies, Wilmington, DE, USA). Total mRNA was retro-transcribed to cDNA using the SuperScript First-Strand Synthesis System kit (Invitrogen, Carlsbad, CA, USA). Twenty-four overlapping RT-PCR primer sets (available on request) generating products of size ranging between 319 and 621 bp were designed to cover the NF1 coding sequence of about 8.6 kb in length (NM 000267.3). PCR reactions were carried out with the Platinum PCR SuperMix High Fidelity kit (Invitrogen) using 2-µL cDNA as templates in a total volume of 50 μL. DNA amplification was achieved by an initial denaturation step at 94 °C for 5 min, followed by 35 cycles of 94 °C for 15 s, 60 °C for 30 s, and 68 °C for 1 min.

#### 4.2.4. Sanger Sequencing

PCR products were purified using the GenUP Exo SAP kit (Biotech Rabbit, Berlin, Germany) and amplified by the Big Dye Terminator Cicle Sequencing kit (Applied Biosystems, Foster City, CA, USA).

#### 4.2.5. Variant Analysis

The *NF1* variants identified through cDNA sequencing or NGS were filtered according to allele frequency <0.001 in gnomAD dataset (https://gnomad.broadinstitute.org, accessed on 7 January 2021), presence in ClinVar (https://www.ncbi.nlm.nih.gov/clinvar/, accessed on 8 January 2021), evaluated according to the conservation of the affected amino acid residues (GERP, http://mendel.stanford.edu/SidowLab/downloads/gerp/, accessed on 8 January 2021), analyzed through in silico tools (SIFT, https://sift.bii.a-star.edu.sg; Poly-Phen2, http://genetics.bwh.harvard.edu/pph2/, accessed on 8 January 2021; CADD, https://cadd.gs.washington.edu, accessed on 8 January 2021; Splice AI, https://pypi.org/project/spliceai/, accessed on 8 January 2021) using the Ensebl Variant Effect Predictor (VEP) pipeline (https://www.ensembl.org/info/docs/tools/vep/index.html, accessed on 8 January 2021), and classified according to the American College of Medical Genetics and Genomics (ACMG) guidelines [70]. In the application of ACMG guidelines, the inheritance status of the variant in an affected individual was also considered (PS2 criteria was considered for de novo variants and PP1 criteria for variants cosegregating in affected family members), when this information was available (Table S2). According to the HGMD specifications (http://www.hgmd.cf.ac.uk/ac/index.php, accessed on 8 January 2021), deletions were indicated as intragenic deletions if they involved 20 bp or less within a *NF1* exon or partial deletions if they involved more than 20 bp, affecting single or multiple *NF1* exons.

### 4.3. Statistical Analysis

No sample size calculations were provided a priori due to the exploratory nature of this study. Demographic and other baseline characteristics of patients were summarized with number and percentages for categorical variables, and with mean and standard deviation and median with range for continuous variables. The investigation of genotype–phenotype correlations was performed on the subpopulation of patients fulfilling the NIH criteria for NF1 diagnosis. Univariate and multivariable logistic regression models were carried out to detect possible association between each clinical feature and genotype. The selection process started with univariate analysis and a *p*-value of less than 0.20 was subsequently defined as a candidate for identifying variables to include in the final multivariable logistic regression model. Adjusted Odds Ratios (AORs) with 95% confidence interval (CI) were reported to show the strength of the associations. All statistical tests were 2-sided, and the significance level (alpha error) was set at 0.05. SPSS (IBM Corp.) v.24 was used for computation.

## 5. Conclusions

Genotype–phenotype correlations will be likely playing a more and more important role in the management of NF1 patients. This is especially true when a variant associated with an increased risk of developing a major clinical manifestation is detected in a patient. For example, our observations suggest that the identification of the stop-gain variant c.6855C>A; p.(Y2285*) would prompt the investigation of endocrinological disorders. Similarly, it would be advisable to investigate the presence of structural brain lesions in patients harboring the stop gain variant c.3721C>T; p.(R1241*) or the presence of skeletal abnormalities in subjects carrying frameshift variants or whole gene deletions. The actual relevance of other interesting though not so straightforward associations, such as the negative correlation between missense variants and neurofibromas, would deserve additional investigations and a further refinement in future studies.

The increasing knowledge on genotype–phenotype correlations in NF1 will hopefully guide the adoption of the most appropriate strategies to implement the management of affected individuals (e.g., organ-specific surveillance), with possible positive implications on the overall prognosis. Although NF1 genetic testing has been usually reserved for uncertain cases, the identification of novel genotype–phenotype correlations is pushing towards a higher demand to allow more targeted clinical interventions. A further highly relevant point is the identification of causative genetic variants in patients not fulfilling the current NIH criteria, thanks to the widespread diffusion of NGS technologies. In light of the increasing number of NF1 patients receiving a genetic diagnosis and considering the relevant role of genetic findings as prognostic guidance, we also suggest that an update of the NIH criteria to ponder the genetic aspects in the diagnostic algorithm would be advisable. Eventually, a better understanding of the contributory role played by modifier genes in determining the NF1 phenotype and the functional effects of genomic variants through advanced RNA analysis techniques represent the main challenges towards the development of a patient-centered genotype–phenotype fingerprint in the next future.

## Figures and Tables

**Table 1:** Baseline characteristics.

			Fulfilling the NIH Criteria for Diagnosis			Genetic Confirmation		
		Total (*n* = 583)	Yes (Group A) (*n* = 365)	No (Group B) (*n* = 218)	*p*	Yes (*n* = 351)	No (*n*= 232)	*p*
Age, years		16.8 ± 11.91 14.0 (1.0–73.0)	18.5 ± 12.86 15.0 (1.0–71.0)	14.1 ± 9.55 12.5 (2.0–73.0)	<0.001 **	17.5 ± 12.59 15.0 (2.0–71.0)	15.7 ± 10.74 14.0 (1.0–73.0)	0.18
Age classes	0–12 years	247 (42.4)	138 (37.8)	109 (50.0)	-	145 (41.3)	102 (44.0)	-
	13–18 years	310 (53.2)	207 (56.7)	103 (47.2)	0.009 **	189 (53.8)	121 (52.2)	0.74
	19–44 years	26 (4.5)	20 (5.5)	6 (2.7)	-	17 (4.8)	9 (3.9)	-
Sex, males		309 (53.0)	182 (49.9)	127 (58.3)	0.049 **	181 (51.6)	128 (55.2)	0.39
Positive family history *		134 (23.0)	104 (28.5)	30 (13.8)	0.001 **	108 (30.8)	26 (11.2)	<0.001 **
Status	De novo	60 (10.3)	48 (13.2)	12 (5.5)	-	60 (17.1)	0 (0.0)	-
	Maternal	39 (6.7)	30 (8.2)	9 (4.1)	<0.001 **	37 (10.5)	2 (0.9)	<0.001 **
	Paternal	48 (8.2)	35 (9.6)	13 (6.0)	-	46 (13.1)	2 (0.9)	-
	Not available	436 (74.8)	252 (69.0)	184 (84.4)	-	208 (59.3)	228 (98.3)	-
Number of NIH criteria satisfied		2.3 ± 1.41 2.0 (1.0–7.0)	3.1 ± 1.21 3.0 (2.0–7.0)	1.0 ± 0.00 1.0 (1.0–1.0)	<0.001 **	2.8 ± 1.40 3.0 (1.0–7.0)	1.6 ± 1.04 1.0 (1.0–7.0)	<0.001 **
Deceased		3 (0.5)	3 (0.8)	0 (0.0)	0.30	3 (0.9)	0 (0.0)	0.16
Genetic confirmation		351 (60.3)	287 (78.6)	64 (29.4)	<0.001 **	-	-	-
Fulfilling NIH criteria for diagnosis		365 (62.6)	-	-	-	287 (81.8)	78 (33.6)	<0.001 **

NF1 = neurofibromatosis type I, NIH = National Institutes of Health. * First-degree relative meeting NIH criteria for NF1 diagnosis. Data are reported as mean with standard deviation, median with range, or count with frequency, as appropriate. ** = *p*-value ≤ 0.05

**Table 2:** Frequency of clinical features.

		Fulfilling the NIH Criteria for Diagnosis			Genetic Confirmation		
	Total (*n* = 583)	Yes (*n* = 365) (Group A)	No (*n* = 218) (Group B)	*p*	Yes (*n* = 351)	No (*n* = 232)	*p*
CALMs	522 (89.5)	351 (96.2)	171 (78.4)	<0.001 **	316 (90.0)	206 (88.8)	0.63
Freckling	280 (48.0)	279 (76.4)	1 (0.5)	<0.001 **	222 (63.2)	58 (25.0)	<0.001 **
Other neurological findings (e.g., abnormal tone/DTRs, tremor, ataxia)	188 (32.1)	136 (37.3)	52 (23.9)	0.001 **	130 (37.0)	58 (25.0)	0.002 **
Neurofibromas (cNFs, sNFs)	137 (23.5)	134 (36.7)	3 (1.4)	<0.001 **	108 (30.8)	29 (12.5)	<0.001 **
Common neurological features †	120 (20.6)	85 (23.3)	35 (16.1)	0.032 **	83 (23.6)	37 (15.9)	0.024 **
OPG	94 (16.1)	87 (23.8)	7 (3.2)	<0.001 **	72 (20.5)	22 (9.5)	0.009 **
Lisch nodules	89 (15.3)	87 (23.8)	2 (0.9)	<0.001 **	74 (21.1)	15 (6.5)	<0.001 **
Scoliosis	89 (15.3)	77 (21.1)	12 (5.5)	<0.001 **	63 (17.9)	26 (11.2)	0.027 **
Neurofibromas (pNFs)	76 (13.0)	75 (20.5)	1 (0.5)	<0.001 **	68 (19.4)	8 (3.4)	<0.001 **
Other skeletal alterations (e.g., osteoporosis, vertebral malformations)	55 (9.4)	44 (12.1)	11 (5.0)	0.005 **	39 (11.1)	16 (6.9)	0.09
Neuroimaging abnormalities (e.g., Chiari malformation, tethered cord)	36 (6.2)	29 (7.9)	7 (3.2)	0.07	24 (6.8)	12 (5.2)	0.84
Endocrinological abnormalities (e.g., thyreopathy, Addison’s disease, PP)	31 (5.3)	24 (6.6)	7 (3.2)	0.08	22 (6.3)	9 (3.9)	0.21
Headache	27 (4.6)	23 (6.3)	4 (1.8)	0.012 **	21 (6.0)	6 (2.6)	0.06
Epilepsy	27 (4.6)	18 (4.9)	9 (4.1)	0.64	18 (5.1)	9 (3.9)	0.48
Gliomas other than OPG (e.g., pilocytic astrocytoma, glioblastoma)	24 (4.1)	22 (6.0)	2 (0.9)	0.008 **	21 (6.0)	3 (1.3)	0.018 **
Non-glioma brain tumors (e.g., amartoma, meningioma, lipoma)	24 (4.1)	15 (4.1)	9 (4.1)	0.66	16 (4.6)	8 (3.4)	0.87
Severe learning disability	17 (2.9)	14 (3.8)	3 (1.4)	0.09	13 (3.7)	4 (1.7)	0.16
Sphenoid bone dysplasia	15 (2.6)	14 (3.8)	1 (0.5)	0.026 **	13 (3.7)	2 (0.9)	0.08
Cerebrovascular abnormalities (ectasia, aneurysms, hypoplasia)	15 (2.6)	15 (4.1)	0 (0.0)	0.005 **	10 (2.8)	5 (2.2)	0.90
Moyamoya syndrome	15 (2.6)	14 (3.8)	1 (0.5)	0.026 **	13 (3.7)	2 (0.9)	0.08
Tibial dysplasia	13 (2.2)	11 (3.0)	2 (0.9)	0.21	11 (3.1)	2 (0.9)	0.08
Noonan-like dysmorphic features	13 (2.2)	8 (2.2)	5 (2.3)	0.95	12 (3.4)	1 (0.4)	0.017 **
Behavioral abnormalities (ADHD, ARFID, ASD)	12 (2.1)	12 (3.3)	0 (0.0)	0.007 **	11 (3.1)	1 (0.4)	0.024 **
Severe DD/ID	12 (2.1)	7 (1.9)	5 (2.3)	0.77	9 (2.6)	3 (1.3)	0.29
Lymphatic and vascular abnormalities (e.g., angioma, cavernoma, LD)	11 (1.9)	8 (2.2)	3 (1.4)	0.48	7 (2.0)	4 (1.7)	0.82
Pectus excavatum	10 (1.7)	10 (2.7)	0 (0.0)	0.013 **	9 (2.6)	1 (0.4)	0.05
Renal malformations/disease (multicystic/duplex kidney, hydronephrosis)	7 (1.2)	3 (0.8)	4 (1.8)	0.17	4 (1.1)	3 (1.3)	0.63
Variants in cerebrovascular anatomy	7 (1.2)	7 (1.9)	0 (0.0)	0.06	5 (1.4)	2 (0.9)	0.55
Hypertension	7 (1.2)	6 (1.6)	1 (0.5)	0.20	5 (1.4)	2 (0.9)	0.55
Ocular abnormalities (coloboma, glaucoma)	4 (0.7)	3 (0.8)	1 (0.5)	0.99	4 (1.1)	0 (0.0)	0.16
Spinal tumors (astrocytoma)	3 (0.5)	2 (0.5)	1 (0.5)	0.99	2 (0.6)	1 (0.4)	0.99
Pulmonic stenosis	3 (0.5)	2 (0.5)	1 (0.5)	0.99	3 (0.9)	0 (0.0)	0.16
Other cardiac abnormalities (valvulopathies, arrhythmia)	3 (0.5)	2 (0.5)	1 (0.5)	0.99	1 (0.3)	2 (0.9)	0.57
Renovascular abnormalities	1 (0.2)	1 (0.3)	0 (0.0)	0.99	0 (0.0)	1 (0.4)	0.34

ADHD = attention deficit-hyperactivity disorder; ARFID = avoidant/restrictive food intake disorder; ASD = autism spectrum disorder; CALMs = café-au-lait macules, cNFs = cutaneous neurofibromas, DD = developmental delay, DTRs = deep tendon reflexes, ID = intellectual disability, LD = lymphatic dysplasia, OPG = optic pathway glioma, pNFs = plexiform neurofibromas, PP = precocious puberty; sNFs = subcutaneous neurofibromas. † headache, epilepsy, behavioral abnormalities, severe learning disabilities, and DD/ID. ** = *p*-value ≤ 0.05.

**Table 3:** Genotype summary (*n* = 351).

			Fulfilling the NIH Criteria for Diagnosis			
		Total (*n* = 351)	Yes (*n* = 287) (Group A)	No (*n* = 64) (Group B)	Univariate	Multivariate
Variant types	Stop-gain	100 (28.5)	87 (30.3)	13 (20.3)	0.11 *	NS
	Missense	85 (24.2)	63 (22.0)	22 (34.4)	0.036 **	NS
	Frameshift	77 (21.9)	66 (23.0)	11 (17.2)	0.31	-
	Splicing	49 (14.0)	38 (13.2)	11 (17.2)	0.41	-
	Whole gene deletion	12 (3.4)	10 (3.5)	2 (3.1)	0.029 **	0.04 (0.01–0.29); 0.002
	Partial deletion	11 (3.1)	8 (2.8)	3 (4.7)	0.001 **	0.02 (0.01–0.14), <0.001
	Intragenic deletion	13 (3.7)	12 (4.2)	1 (1.6)	0.32	-
	Duplication	2 (0.6)	2 (0.7)	0 (0.0)	0.99	-
	Start loss	1 (0.3)	0 (0.0)	1 (1.6)	0.42	-
	Synonymous (exon skipping) †	1 (0.3)	1 (0.3)	0 (0.0)	0.42	-
Recurrent SNVs (NM_001042492.3; NP_001035957.1)	Wild type	23 (6.6)	18 (6.3)	5 (7.8)	0.99	-
	c.574C>T; p.(R192*)	7 (2.0)	7 (2.4)	0 (0.0)	0.20 *	NS
	c.6855C>A; p.(Y2285*)	7 (2.0)	7 (2.4)	0 (0.0)	0.20 *	NS
	c.3721C>T; p.(R1241*)	5 (1.4)	5 (1.7)	0 (0.0)	0.59	-
	c.5488C>T; p.(R1830C) c.6772C>T; p.(R2258*)	5 (1.4) 4 (1.1)	2 (0.7) 4 (1.4)	3 (4.7) 0 (0.0)	0.044 ** 0.20 *	0.14 (0.02–0.87); 0.035 NS
	c.910C>T; p.(R304*)	4 (1.1)	4 (1.4)	0 (0.0)	0.99	-
	c.2041C>T; p.(R681*)	4 (1.1)	3 (1.0)	1 (1.6)	0.56	-
	c.2970_2972delAAT; p.(M992del)	4 (1.1)	4 (1.4)	0 (0.0)	0.99	-
	Miscellaneous (freq ≤ 0.5%)	288 (82.1)	233 (81.2)	55 (85.9)	-	-
MLPA category	Normal	23 (6.6)	21 (7.3)	2 (3.1)	-	-
	Partial deletion	11 (3.1)	8 (2.8)	3 (4.7)	-	-
	Whole gene deletion	12 (3.4)	10 (3.5)	2 (3.1)	-	-
	Duplication	2 (0.6)	2 (0.7)	0 (0.0)	-	-
	Not executed	303 (86.3)	246 (85.7)	57 (89.1)	-	-

Freq = frequency, Indel = insertion/deletion, NGS = Next Generation Sequencing, MLPA = Multiplex Ligation-dependent Probe Amplification. †HGMD ID: CS072236. * = *p*-value ≤ 0.20, therefore included in the multivariate analysis together with ** = *p*-value ≤ 0.05.

**Table 4:** Freckling correlations.

		No (*n* = 65)	Yes (*n* = 222)	Univariate	Multivariate
Age classes	0–12 years	23 (35.4)	92 (41.4)	0.013 **	Ref.
	13–18 years	34 (52.3)	123 (55.4)		NS
	19–44 years	8 (12.3)	7 (3.2)		0.22 (0.07–0.69); 0.010
Sex, males	-	32 (49.2)	110 (49.5)	0.96	-
Family history	-	30 (46.2)	58 (26.1)	0.002 **	0.41 (0.23–0.73); 0.003
Status	De novo	8 (12.3)	40 (18.0)	0.021 **	-
	Maternal	12 (18.5)	17 (7.7)		-
	Paternal	11 (16.9)	23 (10.4)		NS
	Not available	34 (52.3)	142 (64.0)		-
Duplication	-	0 (0.0)	2 (0.9)	0.44	-
Partial deletions	-	2 (3.1)	6 (2.7)	0.87	-
Whole gene deletions	-	0 (0.0)	10 (4.5)	0.08 *	NS
Splicing variants	-	13 (20.0)	25 (11.3)	0.07 *	NS
Missense variants	-	17 (26.2)	46 (20.7)	0.35	-
Stop-gain variants	-	15 (23.1)	72 (32.4)	0.15 *	NS
Frameshift variants	-	16 (24.6)	50 (22.5)	0.72	-
Intragenic deletions	-	2 (3.1)	10 (4.5)	0.61	-
c.574C>T; p.(R192*)	-	0 (0.0)	7 (3.2)	0.15 *	NS
c.6855C>A; p.(Y2285*)	-	1 (1.5)	6 (2.7)	0.59	-
c.3721C>T; p.(R1241*)	-	2 (3.1)	3 (1.4)	0.32	-
c.6772C>T; p.(R2258*)	-	1 (1.5)	3 (1.4)	0.99	-
c.910C>T; p.(R304*)	-	0 (0.0)	4 (1.8)	0.58	-
c.2041C>T; p.(R681*)	-	1 (1.5)	2 (0.9)	0.54	-
c.5488C>T; p.(R1830C)	-	1 (1.5)	1 (0.5)	0.40	-
c.2970_2972delAAT; p.(M992del)	-	1 (1.5)	3 (1.4)	0.99	-

* = *p*-value ≤ 0.20, therefore included in the multivariate analysis, together with ** = *p*-value ≤ 0.05.

**Table 5:** Neurofibromas (pNFs) correlations.

		No (*n* = 219)	Yes (*n* = 68)	Univariate	Multivariate
Age classes	0–12 years	93 (42.5)	22 (32.4)	0.33	-
	13–18 years	115 (52.5)	42 (61.8)		-
	19–44 years	11 (5.0)	4 (5.9)		-
Sex, males	-	107 (48.9)	35 (51.5)	0.71	-
Family history	-	71 (32.4)	17 (25.0)	0.25	-
Status	De novo	40 (18.3)	8 (11.8)	0.10 *	-
	Maternal	23 (10.5)	6 (8.8)		-
	Paternal	30 (13.7)	4 (5.9)		NS
	Not available	126 (57.5)	50 (73.5)		-
Duplication	-	1 (0.5)	1 (1.5)	0.38	-
Partial deletions	-	7 (3.2)	1 (1.5)	0.45	-
Whole gene deletions	-	6 (2.7)	4 (5.9)	0.22	-
Splicing variants	-	28 (12.8)	10 (14.7)	0.68	-
Missense variants	-	57 (26.0)	6 (8.8)	0.003 **	0.28 (0.11–0.67); 0.005
Stop-gain variants	-	58 (26.5)	29 (42.6)	0.011 **	NS
Frameshift variants	-	51 (23.3)	15 (22.1)	0.83	-
Intragenic deletions	-	10 (4.6)	2 (2.9)	0.56	-
c.574C>T; p.(R192*)	-	3 (1.4)	4 (5.9)	0.035 **	NS
c.6855C>A; p.(Y2285*)	-	4 (1.8)	3 (4.4)	0.23	-
c.3721C>T; p.(R1241*)	-	3 (1.4)	2 (2.9)	0.39	-
c.6772C>T; p.(R2258*)	-	3 (1.4)	1 (1.5)	0.95	-
c.910C>T; p.(R304*)	-	3 (1.4)	1 (1.5)	0.95	-
c.2041C>T; p.(R681*)	-	2 (0.9)	1 (1.5)	0.69	-
c.5488C>T; p.(R1830C)	-	2 (0.9)	0 (0.0)	0.43	-
c.2970_2972delAAT; p.(M992del)	-	4 (1.8)	0 (0.0)	0.58	-

pNFs = plexiform neurofibromas. * = *p*-value ≤ 0.20, therefore included in the multivariate analysis together with ** = *p*-value ≤ 0.05.

**Table 6:** Lisch nodules correlations.

		No (*n* = 213)	Yes (*n* = 74)	Univariate	Multivariate
Age classes	0–12 years	93 (43.7)	22 (29.7)	0.002 **	Ref.
	13–18 years	105 (49.3)	52 (70.3)		2.37 (1.31–4.28); 0.004
	19–44 years	15 (7.0)	0 (0.0)		NS
Sex, males	-	108 (50.7)	34 (45.9)	0.48	-
Family history	-	74 (34.7)	14 (18.9)	0.011 **	0.40 (0.21–0.78); 0.007
Status	De novo	34 (16.0)	14 (18.9)	0.13 *	-
	Maternal	25 (11.7)	4 (5.4)		-
	Paternal	29 (13.6)	5 (6.8)		NS
	Not available	125 (58.7)	51 (68.9)		-
Duplication	-	2 (0.9)	0 (0.0)	0.40	-
Partial deletions	-	5 (2.3)	3 (4.1)	0.43	-
Whole gene deletions	-	6 (2.8)	4 (5.4)	0.30	-
Splicing variants	-	28 (13.1)	10 (13.5)	0.94	-
Missense variants	-	46 (21.6)	17 (23.0)	0.81	-
Stop-gain variants	-	64 (30.0)	23 (31.1)	0.87	-
Frameshift variants	-	54 (25.4)	12 (16.2)	0.11 *	NS
Intragenic deletions	-	8 (3.8)	4 (5.4)	0.54	-
c.574C>T; p.(R192*)	-	5 (2.3)	2 (2.7)	0.86	-
c.6855C>A; p.(Y2285*)	-	3 (1.4)	4 (5.4)	0.06 *	6.03 (0.98–36.94); 0.05
c.3721C>T; p.(R1241*)	-	5 (2.3)	0 (0.0)	0.33	-
c.6772C>T; p.(R2258*)	-	3 (1.4)	1 (1.4)	0.99	-
c.910C>T; p.(R304*)	-	3 (1.4)	1 (1.4)	0.99	-
c.2041C>T; p.(R681*)	-	2 (0.9)	1 (1.4)	0.76	-
c.5488C>T; p.(R1830C)	-	2 (0.9)	0 (0.0)	0.99	-
c.2970_2972delAAT; p.(M992del)	-	3 (1.4)	1 (1.4)	0.99	-

* = *p*-value ≤ 0.20, therefore included in the multivariate analysis together with ** = *p*-value ≤ 0.05.

**Table 7:** Skeletal alterations correlations.

		No (*n* = 249)	Yes (*n* = 38)	Univariate	Multivariate
Age classes	0–12 years	98 (39.4)	17 (44.7)	0.28	-
	13–18 years	136 (54.6)	21 (55.3)		-
	19–44 years	15 (6.0)	0 (0.0)		-
Sex, males	-	123 (49.4)	19 (50.0)	0.95	-
Family history (first degree)	-	75 (30.1)	13 (34.2)	0.61	-
Status	De novo	44 (17.7)	4 (10.5)	0.75	-
	Maternal	25 (10.0)	4 (10.5)		-
	Paternal	29 (11.6)	5 (13.2)		-
	Not available	151 (60.6)	25 (65.8)		-
Duplication	-	2 (0.8)	0 (0.0)	0.99	-
Partial deletions	-	7 (2.8)	1 (2.6)	0.95	-
Whole gene deletions	-	7 (2.8)	3 (7.9)	0.11 *	4.09 (0.98–17.08); 0.05
Splicing variants	-	36 (14.5)	2 (5.3)	0.12 *	NS
Missense variants	-	57 (22.9)	6 (15.8)	0.33	-
Stop-gain variants	-	78 (31.3)	9 (23.7)	0.34	-
Frameshift variants	-	51 (20.5)	15 (39.51)	0.010 **	2.81 (1.34–5.87); 0.006
Intragenic deletions	-	10 (4.0)	2 (5.3)	0.72	-
c.574C>T; p.(R192*)	-	6 (2.4)	1 (2.6)	0.99	-
c.6855C>A; p.(Y2285*)	-	7 (2.8)	0 (0.0)	0.60	-
c.3721C>T; p.(R1241*)	-	4 (1.6)	1 (2.6)	0.51	-
c.6772C>T; p.(R2258*)	-	3 (1.2)	1 (2.6)	0.44	-
c.910C>T; p.(R304*)	-	4 (1.6)	0 (0.0)	0.99	-
c.2041C>T; p.(R681*)	-	3 (1.2)	0 (0.0)	0.99	-
c.5488C>T; p.(R1830C)	-	2 (0.8)	0 (0.0)	0.99	-
c.2970_2972delAAT; p.(M992del)	-	3 (1.2)	1 (2.6)	0.44	-

* = *p*-value ≤ 0.20, therefore included in the multivariate analysis together with ** = *p*-value ≤ 0.05.

**Table 8:** Structural brain lesions correlations.

		No (*n* = 244) †	Yes (*n* = 23) †	Univariate	Multivariate
Age classes	0–12 years	100 (41.0)	7 (30.4)	0.29	-
	13–18 years	133 (54.5)	16 (69.6)		-
	19–44 years	11 (4.5)	0 (0.0)		-
Sex, males	-	123 (50.4)	10 (43.5)	0.53	-
Family history (first degree)	-	76 (31.1)	5 (21.7)	0.35	-
Status	De novo	43 (17.6)	4 (17.4)	0.32	-
	Maternal	23 (9.4)	5 (21.7)		-
	Paternal	31 (12.7)	2 (8.7)		-
	Not available	147 (60.2)	12 (52.2)		-
Duplication	-	1 (0.4)	0 (0.0)	0.99	-
Partial deletions	-	8 (3.3)	0 (0.0)	0.99	-
Whole gene deletions	-	10 (4.1)	0 (0.0)	0.99	-
Splicing variants	-	31 (12.7)	4 (17.4)	0.52	-
Missense variants	-	56 (23.0)	3 (13.0)	0.27	-
Stop-gain variants	-	70 (28.7)	9 (39.1)	0.29	-
Frameshift variants	-	57 (23.4)	6 (26.1)	0.77	-
Intragenic deletions	-	10 (4.1)	1 (4.3)	0.99	-
c.574C>T; p.(R192*)	-	6 (2.5)	0 (0.0)	0.99	-
c.6855C>A; p.(Y2285*)	-	6 (2.5)	0 (0.0)	0.99	-
c.3721C>T; p.(R1241*)	-	3 (1.2)	2 (8.7)	0.06 *	7.65 (1.21–48.36); 0.031
c.6772C>T; p.(R2258*)	-	4 (1.6)	0 (0.0)	0.99	-
c.910C>T; p.(R304*)	-	3 (1.2)	0 (0.0)	0.99	-
c.2041C>T; p.(R681*)	-	3 (1.2)	0 (0.0)	0.99	-
c.5488C>T; p.(R1830C)	-	2 (0.8)	0 (0.0)	0.99	-
c.2970_2972delAAT; p.(M992del)	-	3 (1.2)	0 (0.0)	0.99	-

† The total number of patients is *n* = 267, since brain magnetic resonance imaging (MRI) was not available in 20 subjects. * = *p*-value ≤ 0.20.

**Table 9:** Endocrinological abnormalities correlations.

		No (*n* = 267)	Yes (*n* = 20)	Univariate	Multivariate
Age classes	0–12 years	108 (40.4)	7 (35.0)	0.43	Ref.
	13–18 years	144 (53.9)	13 (65.0)		-
	19–44 years	15 (5.6)	0 (0.0)		-
Sex, males	-	135 (50.6)	7 (35.0)	0.18 *	NS
Family history (first degree)	-	85 (31.8)	3 (15.0)	0.12 *	NS
Status	De novo	42 (15.7)	6 (30.0)	0.39	-
	Maternal	28 (10.5)	1 (5.0)		-
	Paternal	32 (12.0)	2 (10.0)		-
	Not available	165 (61.8)	11 (55.0)		-
Duplication	-	2 (0.7)	0 (0.0)	0.99	-
Partial deletions	-	8 (3.0)	0 (0.0)	0.99	-
Whole gene deletions	-	10 (3.7)	0 (0.0)	0.99	-
Splicing variants	-	37 (13.9)	1 (5.0)	0.26	NS
Missense variants	-	58 (21.7)	5 (25.0)	0.73	-
Stop-gain variants	-	79 (29.6)	8 (40.0)	0.33	-
Frameshift variants	-	62 (23.2)	4 (20.0)	0.74	-
Intragenic deletions	-	10 (3.7)	2 (10.0)	0.15 *	NS
c.574C>T; p.(R192*)	-	7 (2.6)	0 (0.0)	0.99	-
c.6855C>A; p.(Y2285*)	-	5 (1.9)	2 (10.0)	0.08 *	5.82 (1.06–32.13); 0.043
c.3721C>T; p.(R1241*)	-	5 (1.9)	0 (0.0)	0.99	-
c.6772C>T; p.(R2258*)	-	4 (1.5)	0 (0.0)	0.99	-
c.910C>T; p.(R304*)	-	4 (1.5)	0 (0.0)	0.99	-
c.2041C>T; p.(R681*)	-	3 (1.1)	0 (0.0)	0.99	-
c.5488C>T; p.(R1830C)	-	2 (0.7)	0 (0.0)	0.99	-
c.2970_2972delAAT; p.(M992del)	-	4 (1.5)	0 (0.0)	0.99	-

* = *p*-value ≤ 0.20.

**Table 10:** Neurofibromas (cNFs and sNFs) correlations.

		No (*n* = 179)	Yes (*n* = 108)	Univariate	Multivariate
Age classes	0–12 years	93 (52.0)	22 (20.4)	<0.001 **	Ref.
	13–18 years	86 (48.0)	71 (65.7)		3.36 (1.91–5.91); <0.001
	19–44 years	0 (0.0)	15 (13.9)		NS
Sex, males	-	91 (50.8)	51 (47.2)	0.55	-
Family history (first degree)	-	59 (33.0)	29 (26.9)	0.28	-
Status	De novo	35 (19.6)	13 (12.0)	0.007 **	-
	Maternal	22 (12.3)	7 (6.5)		-
	Paternal	26 (14.5)	8 (7.4)		NS
	Not available	96 (53.6)	80 (74.1)		-
Duplication	-	2 (1.1)	0 (0.0)	0.53	-
Partial deletions	-	5 (2.8)	3 (2.8)	0.99	-
Whole gene deletions	-	5 (2.8)	5 (4.6)	0.41	-
Splicing variants	-	21 (11.7)	17 (15.7)	0.33	-
Missense variants	-	48 (26.8)	15 (13.9)	0.010 **	0.44 (0.22–0.90); 0.024
Stop-gain variants	-	50 (27.9)	37 (34.3)	0.26	-
Frameshift variants	-	40 (22.3)	26 (24.1)	0.74	-
Intragenic deletions	-	7 (3.9)	5 (4.6)	0.77	-
c.574C>T; p.(R192*)	-	4 (2.2)	3 (2.8)	0.77	-
c.6855C>A; p.(Y2285*)	-	5 (2.8)	2 (1.9)	0.62	-
c.3721C>T; p.(R1241*)	-	2 (1.1)	3 (2.8)	0.37	-
c.6772C>T; p.(R2258*)	-	4 (2.2)	0 (0.0)	0.30	-
c.910C>T; p.(R304*)	-	3 (1.7)	1 (0.9)	0.99	-
c.2041C>T; p.(R681*)	-	2 (1.1)	1 (0.9)	0.99	-
c.5488C>T; p.(R1830C)	-	1 (0.6)	1 (0.9)	0.99	-
c.2970_2972delAAT; p.(M992del)	-	3 (1.7)	1 (0.9)	0.99	-

** = *p*-value ≤ 0.05.

## Data Availability

The data that support the findings of this study are available in the article and the Supplementary Materials.

## Supplementary Materials

The following are available online at https://www.mdpi.com/article/10.3390/cancers13081879/s1, Table S1: Summary of the genetic, clinical, and radiological features of the studied cohort, Table S2: Summary of the genetic corerelations. Table S3: Common neurological features† correlations, Table S4: OPGs correlations, Table S5: Scoliosis correlations, Table S6. CALMs correlations, Table S7: Other neurological findings correlations.
