# Supplementary material for: Genotype-Phenotype Correlations in Neurofibromatosis Type 1: A Single-Center Cohort Study
Source: Cancers (Basel). 2021 Apr 14;13(8):1879. doi: 10.3390/cancers13081879 (PMC8070780; doi:10.3390/cancers13081879)
Supplement: Supplementary file 1 [file cancers-13-01879-s001.zip › cancers-1153713-supplementary.pdf]

cancers

MDPI

Supplementary

Genotype-Phenotype Correlations in Neurofibromatosis Type 1: A Single-Center Cohort Study

Marcello Scala et. al

Table S3. Common neurological features† correlations.

		No (*N* = 214)	Yes (*N* = 73)	Univariate	Multivariate
Age classes	0–12 years	86 (40.2)	29 (39.7)	0.52	
	13–18 years	115 (53.7)	42 (57.5)		
	19–44 years	13 (6.1)	2 (2.7)		
Sex, males		106 (49.5)	36 (49.3)	0.97	
Family history		70 (32.7)	18 (24.7)	0.20*	NS
Status	De novo	33 (15.4)	15 (20.5)	0.57	
	Maternal	21 (9.8)	8 (11.0)		
	Paternal	28 (13.1)	6 (8.2)		
	Not available	132 (61.7)	44 (60.3)		
Duplication		1 (0.5)	1 (1.4)	0.42	
Partial deletions		5 (2.3)	3 (4.1)	0.43	
Whole gene deletions		7 (3.3)	3 (4.1)	0.74	
Splicing variants		29 (13.6)	7 (9.6)	0.38	
Missense variants		49 (22.9)	14 (19.2)	0.51	
Stop gain variants		66 (30.8)	19 (26.0)	0.44	
Frameshift variants		47 (22.0)	23 (31.5)	0.10*	NS
Intragenic deletions		9 (4.2)	1 (1.4)	0.25	
c.574C>T; p.(R192*)		6 (2.8)	1 (1.4)	0.49	
c.6855C>A; p.(Y2285*)		6 (2.8)	1 (1.4)	0.49	
c.3721C>T; p.(R1241*)		5 (2.3)	0 (0.0)	0.19*	NS
c.6772C>T; p.(R2258*)		4 (1.9)	0 (0.0)	0.58	
c.910C>T; p.(R304*)		3 (1.4)	1 (1.4)	0.99	
c.2041C>T; p.(R681*)		3 (1.4)	0 (0.0)	0.57	
c.5488C>T; p.(R1830C)		2 (0.9)	0 (0.0)	0.41	

† Common neurological features include: headache, epilepsy, behavioral abnormalities, severe learning disabilities and DD/ID. * = *p*-value ≤ 0.20, therefore included in the multivariate analysis together with ** = *p*-value ≤ 0.05.

Table S4. OPGs correlations.

Column Title	Column Title	No (*N* = 197)	Yes (*N* = 70)	Univariate	Multivariate
Age classes	0–12 years	79 (40.1)	28 (40.0)		
	13–18 years	107 (54.3)	42 (60.0)	0.12*	NS
	19–44 years	11 (5.6)	0 (0.0)		
Sex, males		102 (51.8)	31 (44.3)	0.28	
Family history (first degree)		57 (28.9)	24 (34.3)	0.40	
Status	De novo	31 (15.7)	16 (22.9)		
	Maternal	21 (10.7)	7 (10.0)	0.33	
	Paternal	22 (11.2)	11 (15.7)		
	Not available	123 (62.4)	36 (51.4)		
Duplication		0 (0.0)	1 (1.4)	0.26	
Partial deletions		6 (3.0)	2 (2.9)	0.94	
Whole gene deletions		7 (3.6)	3 (4.3)	0.78	
Splicing variants		23 (11.7)	10 (14.3)	0.57	
Missense variants		49 (24.9)	10 (14.3)	0.07*	NS
Stop gain variants		52 (26.4)	25 (35.7)	0.14*	NS
Frameshift variants		52 (26.4)	15 (21.4)	0.41	
Intragenic deletions		5 (2.5)	4 (5.7)	0.21	
c.574C>T; p.(R192*)		6 (3.0)	0 (0.0)	0.35	
c.6855C>A; p.(Y2285*)		6 (3.0)	0 (0.0)	0.35	
c.3721C>T; p.(R1241*)		2 (1.0)	3 (4.3)	0.12*	NS
c.6772C>T; p.(R2258*)		3 (1.5)	1 (1.4)	0.99	
c.910C>T; p.(R304*)		1 (0.5)	2 (2.9)	0.17*	NS
c.2041C>T; p.(R681*)		3 (1.5)	0 (0.0)	0.57	
c.5488C>T; p.(R1830C)		2 (1.0)	0 (0.0)	0.99	

OPGs = optic pathway gliomas. * = *p*-value ≤ 0.20, therefore included in the multivariate analysis together with ** = *p*-value ≤ 0.05.

Table S5. Scoliosis correlations.

Column Title	Column Title	No (N = 228)	Yes (N = 59)	Univariate	Multivariate
Age classes	0–12 years	105 (46.1)	10 (16.9)		Ref.
	13–18 years	108 (47.4)	49 (83.1)	<0.001**	4.76 (2.29–9.90); <0.001
	19–44 years	15 (6.6)	0 (0.0)		NS
Sex, males		118 (51.8)	24 (40.7)	0.13*	NS
Family history (first degree)		74 (32.5)	14 (23.7)	0.21	
Status	De novo	38 (16.7)	10 (16.9)		
	Maternal	23 (10.1)	6 (10.2)	0.99	
	Paternal	27 (11.8)	7 (11.9)		
	Not available	140 (61.4)	36 (61.0)		
Duplication		1 (0.4)	1 (1.7)	0.37	
Partial deletions		7 (3.1)	1 (1.7)	0.57	
Whole gene deletions		6 (2.6)	4 (6.8)	0.12*	NS
Splicing variants		27 (11.8)	9 (15.3)	0.48	
Missense variants		50 (21.9)	13 (22.0)	0.99	
Stop gain variants		68 (29.8)	17 (28.8)	0.88	
Frameshift variants		59 (25.9)	11 (18.6)	0.25	
Intragenic deletions		8 (3.5)	2 (3.4)	0.97	
c.574C>T; p.(R192*)		5 (2.2)	2 (3.4)	0.60	
c.6855C>A; p.(Y2285*)		6 (2.6)	1 (1.7)	0.68	
c.3721C>T; p.(R1241*)		4 (1.8)	1 (1.7)	0.99	
c.6772C>T; p.(R2258*)		4 (1.8)	0 (0.0)	0.59	
c.910C>T; p.(R304*)		4 (1.8)	0 (0.0)	0.59	
c.2041C>T; p.(R681*)		1 (0.4)	2 (3.4)	0.11*	NS
c.5488C>T; p.(R1830C)		2 (0.9)	0 (0.0)	0.99	

* = p-value ≤ 0.20, therefore included in the multivariate analysis together with ** = p-value ≤ 0.05.

Table S6. CALMs correlations.

Column Title	Column Title	No (N = 12)	Yes (N = 275)	Univariate	Multivariate
Age classes	0–12 years	3 (25.0)	112 (40.7)		Ref.
	13–18 years	9 (75.0)	148 (53.8)	0.32	
	19–44 years	0 (0.0)	15 (5.5)		
Sex, males		7 (58.3)	135 (49.1)	0.53	
Family history (first degree)		10 (83.3)	78 (28.4)	<0.001**	0.06 (0.01 - 0.32); 0.001
Status	De novo	1 (8.3)	47 (17.1)		
	Maternal	4 (33.3)	25 (9.1)	0.002**	
	Paternal	4 (33.3)	30 (10.9)		NS
	Not available	3 (25.0)	173 (62.9)		
Duplication		0 (0.0)	2 (0.7)	0.99	
Partial deletions		0 (0.0)	8 (2.9)	0.99	
Whole gene deletions		0 (0.0)	10 (3.6)	0.99	
Splicing variants		2 (16.7)	34 (12.4)	0.66	
Missense variants		1 (8.3)	62 (22.5)	0.47	
Stop gain variants		4 (33.3)	81 (29.5)	0.75	

Frameshift variants	4 (33.3)	66 (24.0)	0.50	
Intragenic deletions	1 (8.3)	9 (3.3)	0.35	
c.574C>T; p.(R192*)	0 (0.0)	7 (2.5)	0.99	
c.6855C>A; p.(Y2285*)	0 (0.0)	7 (2.5)	0.99	
c.3721C>T; p.(R1241*)	1 (8.3)	4 (1.5)	0.19*	NS
c.6772C>T; p.(R2258*)	1 (8.3)	3 (1.1)	0.16*	NS
c.910C>T; p.(R304*)	0 (0.0)	4 (1.5)	0.99	
c.2041C>T; p.(R681*)	0 (0.0)	3 (1.1)	0.99	
c.5488C>T; p.(R1830C)	0 (0.0)	2 (0.7)	0.99	

CALMs = cafè-au-lait macules. * = *p*-value ≤ 0.20, therefore included in the multivariate analysis together with ** = *p*-value ≤ 0.05.

Table S7. Other neurological findings† correlations.

**Column Title**	**Column Title**	**No (*N* = 177)**	**Yes (*N* = 110)**	**Univariate**	**Multivariate**
Age classes	0–12 years	71 (40.1)	44 (40.0)		
	13–18 years	94 (53.1)	63 (57.3)	0.31	
	19–44 years	12 (6.8)	3 (2.7)		
Sex, males		89 (50.3)	53 (48.2)	0.73	
Family history (first degree)		53 (29.9)	35 (31.8)	0.74	
Status	De novo	25 (14.1)	23 (20.9)		
	Maternal	15 (8.5)	14 (12.7)	0.18*	
	Paternal	20 (11.3)	14 (12.7)		NS
	Not available	117 (66.1)	59 (53.6)		
Duplication		2 (1.1)	0 (0.0)	0.53	
Partial deletions		7 (4.0)	1 (0.9)	0.13*	NS
Whole gene deletions		7 (4.0)	3 (2.7)	0.58	
Splicing variants		23 (13.0)	13 (11.8)	0.77	
Missense variants		41 (23.2)	22 (20.0)	0.53	
Stop gain variants		52 (29.4)	33 (30.0)	0.91	
Frameshift variants		39 (22.0)	31 (28.2)	0.24	
Intragenic deletions		5 (2.8)	5 (4.5)	0.44	
c.574C>T; p.(R192*)		6 (3.4)	1 (0.9)	0.19*	NS
c.6855C>A; p.(Y2285*)		5 (2.8)	2 (1.8)	0.59	
c.3721C>T; p.(R1241*)		2 (1.1)	3 (2.7)	0.38	
c.6772C>T; p.(R2258*)		1 (0.6)	3 (2.7)	0.16*	NS
c.910C>T; p.(R304*)		2 (1.1)	2 (1.8)	0.64	
c.2041C>T; p.(R681*)		2 (1.1)	1 (0.9)	0.99	
c.5488C>T; p.(R1830C)		2 (1.1)	0 (0.0)	0.53	

† Other neurological findings include: abnormal muscle tone, abnormal deep tendon reflexes, ataxia, tremor, fatigue, lower limbs pain, hallucinations, neurogenic bladder, hyperkinetic movements, paresthesias, and stereotyped movements. * = *p*-value ≤ 0.20, therefore included in the multivariate analysis together with ** = *p*-value ≤ 0.05.
